# Facile and efficient preparation of high-quality black phosphorus quantum dot films for sensing applications

**DOI:** 10.1039/c9ra10900e

**Published:** 2020-04-01

**Authors:** Yan Zhao, Jie Huang, Ran Zhang, Yinzhou Yan, Yongzhe Zhang

**Affiliations:** Beijing Engineering Research Center of Laser Technology, Institute of Laser Engineering, Beijing University of Technology Beijing 100124 China zhaoyan@bjut.edu.cn; Key Laboratory of Trans-scale Laser Manufacturing Technology, Ministry of Education, Institute of Laser Engineering, Beijing University of Technology Beijing 100124 China; College of Materials Science and Engineering, Beijing University of Technology Beijing 100124 China

## Abstract

In this study, black phosphorus quantum dots (BPQDs) are synthesized by improved ultrasonic-assisted liquid exfoliation. The as-prepared BPQDs are deposited on large-area conductive substrates by electrophoretic deposition, and exhibit high-sensitivity humidity sensing and excellent electrical properties. The results offer not only a facile and efficient method to synthesise stable BPQDs films, but also a new opportunity to develop humidity sensors and nano-electronic devices.

## Abstract 2

In this study, black phosphorus quantum dots (BPQDs) are synthesized by improved ultrasonic-assisted liquid exfoliation.

## Graphical abstract

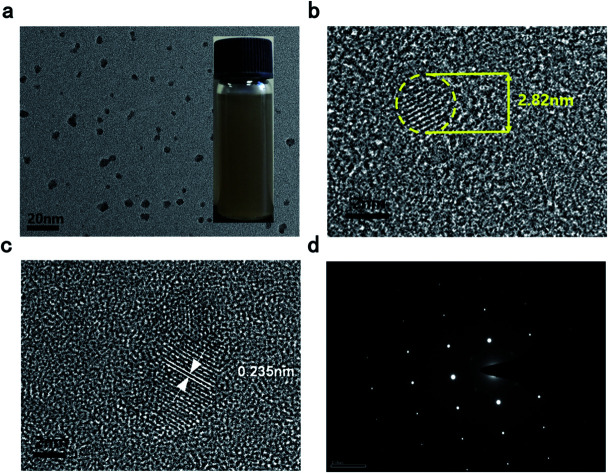

## 1. Introduction

In the past decade, layered two-dimensional (2D) materials such as graphene, hexagonal boron nitride, and transition-metal dichalcogenides (TMDs) have attracted considerable attention because of their formidable physical and structural properties.^1^ Recently, atomically thin black phosphorus (BP) nanosheets have been successfully exfoliated from bulk BP crystals and fabricated into field-effect transistors (FETs).^2^ Thus far, BP has attracted considerable attention with respect to its physical, chemical, and biological properties. In contrast to graphene, BP is a 2D semiconductor with a sufficiently large tunable band gap,^3^ high carrier mobility,^4^ and remarkable in-plane anisotropic properties.^5^ These properties make BP a promising candidate for various applications, including optoelectronic devices,^6–8^ energy storage,^9–11^ gas sensors,^12,13^ and thermoelectric applications.^14^

Numerous bottom-up synthesis methods, such as chemical vapor deposition using metallic catalysts,^15–19^ an epitaxial process,^20–22^ hydrothermal methods,^23^ and reduction methods,^24,25^ have been reported for 2D materials, even for ternary or heterostructure growth.^26–32^ These methods require complex, expensive equipment and harsh conditions without exceptions. Unfortunately, the synthesis of atomic-scale BP has not been adequately investigated because of its complicated chemistry as well as the instability of phosphorus. Efforts combining materials science and chemistry expertise should be devoted to the development of large-scale synthesis methods for BP thin films at the wafer scale, which can lead to additional applications, such as for other 2D materials such as graphene.^33^ Furthermore, it is also necessary to develop methods for the synthesis of large-area, high-quality thin films where the anisotropic properties of BP can be explored on a large scale.^34^ Recently, Li *et al.* have reported thin-film BP with a thickness of 40 nm on a flexible substrate by the deposition of a red phosphorus thin film on a flexible polyester substrate, followed by its conversion into BP using a high-pressure multi-anvil cell at room temperature.^35^ In addition, an amorphous BP film was successfully grown on Si/SiO_2_ or graphene/Cu substrates by pulsed laser deposition using a BP crystal as the precursor,^36^ affording poorly crystallized films with a thickness ranging from several nanometers to tens of nanometers.

Meanwhile, humidity sensors are attracting considerable attention in industrial and environmental fields. 2D materials such as graphene and TMDs have been widely examined for humidity sensing applications because of their high surface-to-volume ratios and excellent mechanical properties.^37–41^ Previously reported studies on BP demonstrate the ambient instability of the devices fabricated by atomically thin flakes;^42–45^ hence, micron-scale BP films have attracted considerable interest in the field of humidity sensors.^46,47^ However, the conventional method for preparing BP films requires high temperature and pressure,^48,49^ the film will be uneven,^50–52^ otherwise, the sensitivity performance of two-dimensional nanomaterial film humidity sensor needs to be improved.^53,54^

In this study, compared to traditional methods, electrophoretic deposition (EPD) was employed to synthesize high-quality BP quantum dot (BPQD) thin films at room temperature and atmospheric pressure. This simple method exhibits advantages of easy operation, cost-effectiveness, environmentally friendly nature, without the use of harsh conditions such as vacuum or inert atmosphere and high temperature and pressure. These BPQD film shows higher sensitivity in humidity detection than other thin film sensors. Impedance spectroscopy and electrical characterization revealed that the sensing mechanism of the BP film sensors is based on modulation of the leakage ionic current.

## 2. Results and discussion

**Fig. 1:**
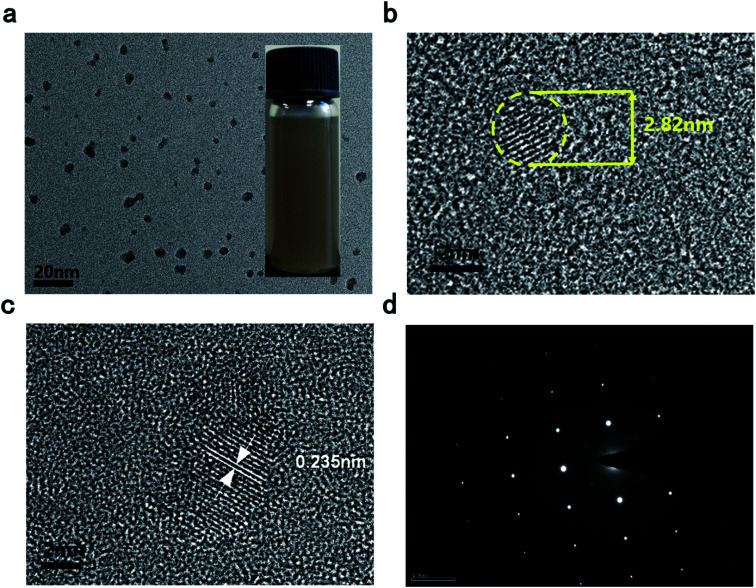
(a) TEM image (inset is the photograph of as-prepared BPDQs) (b and c) HR-TEM image (d) the selected-area electron diffraction (SAED) pattern of BPQDs.

Simple liquid exfoliation was utilized to prepare a highly dispersed suspension of ultra-small BPQDs with a lateral size of ∼10 nm. First, 30 mg of BP powder, which was weighed using an electronic balance, was dispersed into 100 mL of an isopropanol (IPA) solution cooled to 4° in a refrigerator, followed by ultrasonication (40 kHz) for 2 h in an ice water bath. Meanwhile, an ice block was added every 30 min, and the container was overturned every 1 min to completely exfoliate the BP powder and make a uniform, stable dispersion. The as-prepared BPQDs–IPA was stored in a sealed container. Fig. 1(a–c) shows the transmission electron microscopy (TEM) images of BPQDs. Statistical analysis based on TEM measurements was carried out to calculate the precise average diameter of BPQD samples: the QDs exhibited different diameters, with the predominant diameters ranging from 2.82 to ∼10 nm. The interlayer distance of BPQDs was statistically 0.235 nm. Uniform, stable BPQDs were synthesized by ultrasonic-assisted liquid exfoliation in an ice bath. Temperature and ultrasonic waves are well known to inhibit the agglomeration of BPQDs. In addition, uniform, small QDs lead to flat, smooth films. Furthermore, as shown in the high-resolution TEM image at a scale bar of 20 nm (Fig. 1a), a dense, disordered morphology without a long-range order was apparently observed at the interface between the grown film and substrate. This result indicated that BPQDs exhibit an ordered structure with an observed layered structure. Moreover, the selected-area electron diffraction (SAED) image showed a lattice pattern (Fig. 1d). These results further indicated that the obtained BPQDs exhibit perfect lattice structures.

**Fig. 2:**
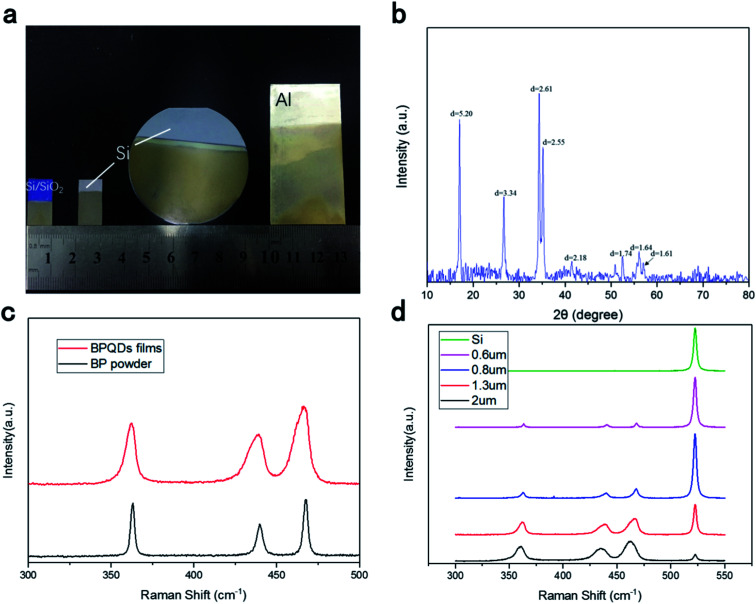
(a) Photographs of samples prepared by electrophoretic deposition on different substrates (from left to right: Si/SiO_2_, Si, Al). (b) XRD results of BPQD films. (c) Raman spectra of 800 nm-thick BPQD films and of BP powder. (d) Raman spectra of the BPQD films with different thicknesses and Si substrate.

BPQDs were deposited on a silicon substrate by EPD. BPQDs with diameters ranging from 3 to 10 nm, which were prepared by liquid exfoliation, were used. Mixtures of magnesium nitrate (Mg(NO_3_)_2_) and IPA at different volume ratios were used as the suspension solvent, and 0.2 mg mL^−1^ of BPQDs was dispersed into the solvent, after ultrasonication for 30 min, affording a stable suspension. The cathode and anode were connected to a silicon wafer and graphite electrode, respectively. The two electrodes were maintained parallel to each other at a distance of 30 mm in the suspension, and a constant deposition voltage of 110 V was used for EPD. X-ray diffraction (XRD) and TEM were utilized to examine the structural properties of BPQD films: a perfect crystal structure and clearly identified phases were observed for the BPQD films (Fig. 2b). Fig. 2c shows the Raman spectra of BPQDs films: A^1^_g_ (out-of-plane), B_2g_, and A^2^_g_ (in-plane) Raman peaks were observed at 362, 438, and 467 cm^−1^, respectively, for BPQD films prepared by EPD. For bulk samples, Raman peaks corresponding to the A^1^_g_, B_2g_, and A^2^_g_ modes were observed at 363, 437, and 468 cm^−1^, respectively. An extremely similar distribution of the peak positions between the films and bulk was observed,^55^ indicative of the good crystalline quality of the sample after EPD. Meanwhile, thin films of different thicknesses, *i.e.*, ranging from 0.3 to 5 μm, were also prepared by adjusting the parameters. The thickness of films was measured by the optical profiler of WykoNT1100 (Veeco). We can see that the Raman peak of the black phosphorus quantum dot film is wider than the bulk black phosphorus, because the black phosphorus quantum dot film has a different structure from the bulk black phosphorus. The bulk black phosphorus lattice structure is single and stable, with a fixed bond angle and bond length. Therefore, the position of its Raman characteristic peak is determined. Black phosphorus quantum dots are formed in clusters. There is mechanical strain between black phosphorus clusters of different sizes. As the thickness of the black phosphorus quantum dot film increases, small black phosphorus quantum dot clusters will gradually for large-scale transitions. Different sizes of black phosphorus quantum dots exhibit different mechanical strain, thereby changing the bond length and bond angle of their physical structure to a small extent, making them show a small difference in Raman peak positions. Raman peaks become wider due to the coexistence of black phosphorus quantum dot clusters of different sizes. With increasing thickness, the peak intensity relatively increased. The BPQD–IPA concentration played a major role in the film thickness, in addition to deposition time and voltage. Moreover, the scale of the films can be regulated and controlled by changing the electrode size. In contrast to the conventional method, the method developed herein can be used for the facile control of the thickness and scale of the films at room temperature and atmospheric pressure.^48,49^

**Fig. 3:**
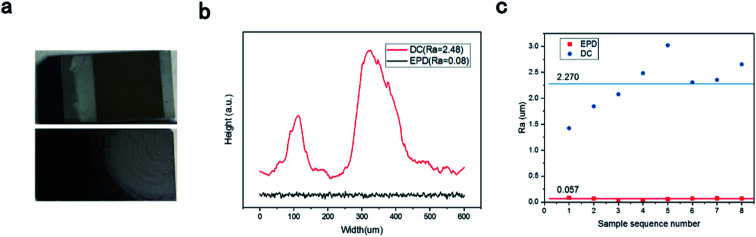
(a) Photographs of samples prepared by electrophoretic deposition (EPD, top) and the drop-coating method (DC, bottom). (b) Cross-section profile and (c) arithmetic mean deviation of surface roughness (*R*_a_) of the BPQD films prepared by EPD and DC.

Clearly, the film surface prepared by drop-coating was not smooth because of the coffee-ring effect (Fig. 3a). A flat, smooth cross-section profile for the BPQD films was observed (Fig. 3(b)). This method leads to significant improvement in the film surface smoothness and the decrease in the dynamic friction coefficient. This feature revealed the long-term stability of the BPQD films used in optoelectronic devices. This system improves the measurement precision *via* the average of the testing values from repeated measurements. The roughness of the BPQDs prepared by EPD (*R*_a_ = 0.06 um) was superior to that prepared by the drop-coating method (*R*_a_ = 0.23 um, Fig. 3(c)).

**Fig. 4:**
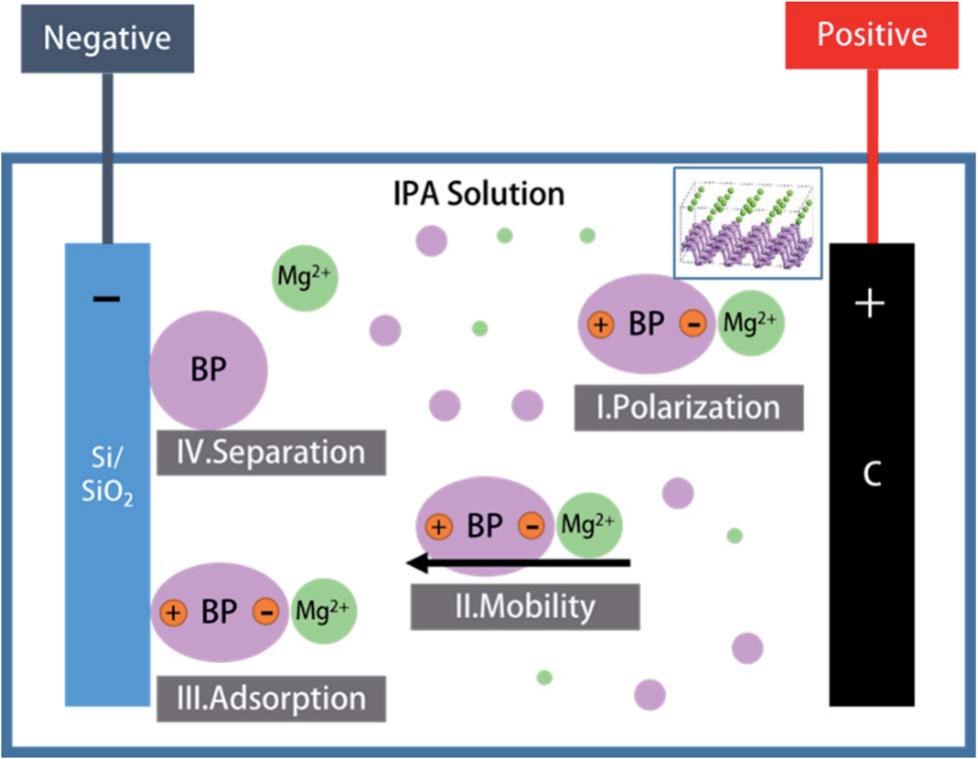
Mechanism for the synthesis of BPQD films by electrophoretic deposition.

After a series of confirmatory experiments, the mechanism for the synthesis of BPQD films by EPD is thought to occur by the following four processes (Fig. 2): (1) Polarization: magnesium nitrate (Mg(NO_3_)_2_) ionizes in the IPA solution, affording free magnesium ions (Mg^2+^). In the BP structure, one phosphorus with five valence electrons provides three electrons for a covalent bond with the other three. Hence, two outermost electrons are free. BPQDs easily adsorb Mg^2+^ because of its high surface area, affording coordinate covalent bonds with Mg^2+^ (Fig. 4). [BP Mg]^2+^ polarizes under an electric field. (2) Mobility: under an electric field, positively charged particles in a colloid shift to the cathode, while negative particles shift to the anode. Therefore, [BP Mg]^2+^ shifts to the cathode. (3) Adsorption: similar to the repulsion of electric charges, unlike charges attract each other. [BP Mg]^2+^ groups are adsorbed on the cathode surface. (4) Separation: as a result of charge transfer, BP groups undergo depolarization after their contact with the cathode, which are separated from Mg^2+^. At length, BPQDs were deposited on the Si/SiO_2_ substrate.

Fig. 4(a) shows the structure of the BPQD-film humidity sensor. The silicon substrate was modified with thin aluminum interdigital electrodes by utilizing vacuum evaporate plating technology. This structure leads to the increased sensitivity of the BPQD film humidity sensor because that more influences of interface state were introduced due to the interdigital electrode configuration. The distance between the electrodes was 0.5 mm. Interdigital electrodes separated by a small distance can lead to the decreased modulation voltage and effectively improved tunability. Fig. 4(b) shows the current–voltage (*I*–*V*) characteristics of the BPQD film sensor: With increasing humidity from 20% to 90%, the *I*–*V* incline increased. Fig. 4(c) shows a series of sensing resistance data as a function of the relative humidity (RH) at room temperature. Specifically, with the increase in RH from 20% to 90%, the resistance of the sensor decreased by ∼4 orders of magnitude. BPQDs with a high specific surface area will easily adsorb water molecules from air, leading to autoionization on the BPQD film surface, and the dissociation of the hydrogen–oxygen bond, affording protons (H^+^) from water molecules, and formed hydroxide ion (OH^−^). This released proton combines with another water molecule to form a hydrated cation (H_3_O^+^). In a low-humidity environment (20%), a small amount of water molecules can adhere to the BPQD film surface, implying the formation of a small amount of H_3_O^+^ and a lower electrical conductivity for the BPQD films. On the other hand, at a higher humidity of 90%, the films exhibited higher electrical conductivity, related to the presence of a high amount of H_3_O^+^.

The calculation formula for the humidity sensitivity of the black phosphorus quantum dot film humidity sensor is as follows:
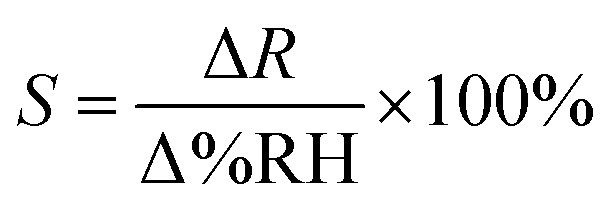


The sensitivity in the formula is defined as the ratio of the change in resistance to the change in relative humidity. The sensitivity value in this paper is calculated to be approximately equal to 1150.

## 3. Conclusions

In conclusion, BPQDs with a lateral size of ∼10 nm was prepared by simple liquid exfoliation. Furthermore, high-sensitivity, stable humidity detection was investigated using the BPQD thin films prepared by electrophoretic deposition. In contrast to the conventional method, the method developed herein can lead to the facile control of the thickness and scale of the films at room temperature and atmospheric pressure. This flat BPQD film with long-term stability demonstrates immense potential for optoelectronic devices, energy storage, gas sensors, and thermoelectric applications.

## 4. Experimental section

### Syntheses and characterization of BPQDs

A highly dispersed suspension of ultra-small BPQDs with a lateral size of ∼10 nm was prepared by simple liquid exfoliation. First, 30 mg of black phosphorus powder, which was weighed using an electronic balance, was dispersed in 100 mL of an isopropanol solution cooled to 4° in a refrigerator, followed by ultrasonication (40 kHz) for 2 h in an ice water bath. Next, an ice block was added every 30 min, the and the container was overturned after 1 min each to completely exfoliate the BP powder and make a stable, uniform dispersion. The as-prepared BPQD–IPA was stored in a sealed container.

### Deposition of BPQD films by electrophoretic deposition

BP quantum dots were deposited on the silicon substrate by electrophoretic deposition. BPQDs with diameters ranging from 3 to 10 nm, which were prepared by liquid exfoliation, were used. Mixtures of magnesium nitrate (Mg(NO_3_)_2_) and IPA with different volume ratios were used as the suspension solvent, and 0.2 mg mL^−1^ of BPQDs was dispersed into the solvent, after ultrasonication for 30 min, to form a stable suspension. The cathode and anode were connected to a silicon wafer and graphite electrode, respectively. The two electrodes were maintained parallel to each other at a distance of 30 mm in the suspension, and a constant deposition voltage of 110 V was used.

### Characterization of BPQDs and BPQDs films

The amorphous phase was characterized by X-ray diffraction (Rigaku Smartlab, Japan) with Cu Kα radiation (*λ* = 1.5406 Å). Transmission electron microscopy (JEOL JEM 2400, Japan) was employed to examine the detailed microstructures and chemical compositions. Cross-sectional TEM specimens were prepared by the application of FIB (JEOL JIB-4500) milling and a lift-off technique. The samples thus obtained were transferred on a copper grid for TEM characterization. The thickness of BPQD films and surface morphology were determined by atomic force microscopy (Park XE-100 AFM). Raman spectra were recorded on a Raman system (T64000 JHON YVORN) equipped with a 532 nm laser source.

### Fabrication and electrical measurements of the BPQD film humidity sensor

**Fig. 5:**
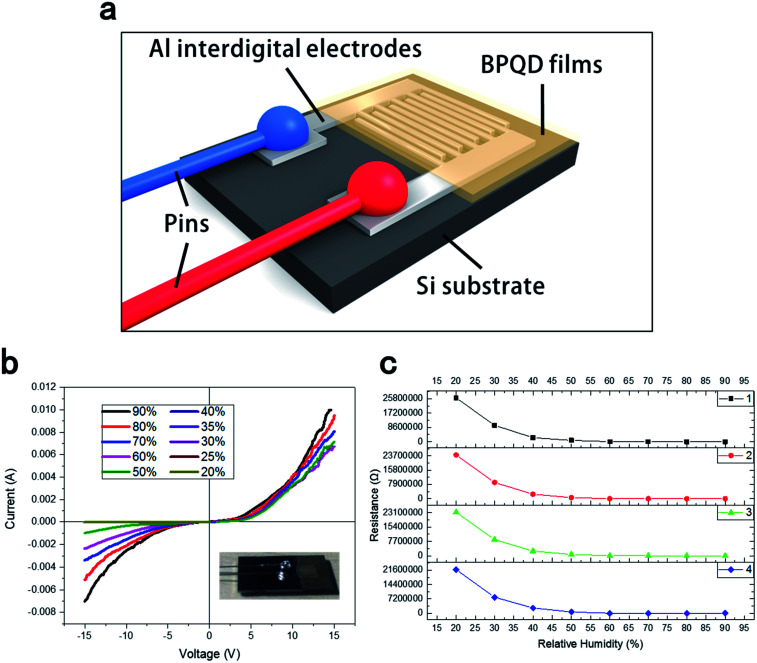
(a) Structure of the BPQD-film humidity sensor. (b) Current–voltage (*I*–*V*) characteristics of the BPQD-film humidity sensor. (c) Sensor resistance as a function of the relative humidity (RH) of the sensor after four tests.

The silicon substrate was modified with thin aluminum interdigital electrodes by utilizing vacuum evaporate plating technology. This structure increases the sensitivity of the BPQD-film humidity sensor because that more influences of interface state were introduced due to the interdigital electrode configuration. The distance between the electrodes was 0.5 mm. Interdigital electrodes with a small distance can decrease the modulation voltage and effectively improve tunability (Fig. 5).

## Conflicts of interest

There are no conflicts to declare.

## Supplementary Material
